# Metabolomics: A suitable foodomics approach to the geographical origin traceability of Ethiopian Arabica specialty coffees

**DOI:** 10.1002/fsn3.3434

**Published:** 2023-05-24

**Authors:** Makiso Urugo Markos, Yetenayet Tola, Biniam T. Kebede, Onwuchekwa Ogah

**Affiliations:** ^1^ Department of Food Science and Postharvest Technology, College of Agricultural Sciences Wachemo University Hosanna Ethiopia; ^2^ Department of Postharvest Management, College of Agriculture and Veterinary Medicine Jimma University Jimma Ethiopia; ^3^ Department of Food Science University of Otago Dunedin New Zealand; ^4^ Department of Biotechnology Ebonyi State University Abakaliki Nigeria

**Keywords:** *Coffee arabica*, foodomics, geographical origin, metabolomics, traceability

## Abstract

*Coffee arabica*, originated in Ethiopia, is considered a quality bean for its high sensory qualities, and has a special price in the world coffee market. The country is a pool of genetic diversity for Arabica coffee, and coffee from different regions has a distinct flavor profile. Their exceptional quality is attributed to their genetic diversity, favorable environmental conditions, and agroforestry‐based production system. However, the country still needs to benefit from its single‐origin product due to a lack of appropriate traceability information to register for its geographical indication. Certification of certain plants or plant‐derived products emerged to inform consumers about their exceptional qualities due to their geographical origin and protect the product from fraud. The recently emerging foodomics approaches, namely proteomics, genomics, and metabolomics, are reported as suitable means of regional agri‐food product authentication and traceability. Particularly, the metabolomics approach provides truthful information on product traceability. Despite efforts by some researchers to trace the geographical origin of Ethiopian Arabica coffees through stable isotope and phenolic compound profiling and elemental analysis, foodomics approaches are not used to trace the geographical origin of Arabica specialty coffees from various parts of the country. A metabolomics‐based traceability system that demonstrates the connection between the exceptional attributes of Ethiopian Arabica specialty coffees and their geographic origin is recommended to maximize the benefit of single‐origin coffees.

## Graphical abstract

Ethiopian Arabica specialty coffee quality. Science of foodomics in geographical origin traceability.

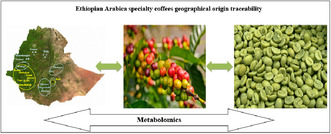

## 1 INTRODUCTION

Coffee, next to petroleum, is the most widely consumed and traded commodity internationally (ICO, 2015; Núñez et al., 2020). *Coffea arabica* (Arabica) and *Coffea canephora* (Robusta) are the two most dominant coffee species with economic and commercial importance globally (Nunez et al., 2021; Núñez et al., 2020). Mainly, *Coffea arabica*, which originated in Ethiopia, accounts for 70% of the world's coffee production, and it is considered a quality bean for its high sensory properties and has a special price in the world coffee market (Adronikos, 2019; Belete et al., 2015; Girmay et al., 2019; Núñez et al., 2020; Scholz et al., 2016). Ethiopia is the world's fifth top Arabica coffee producer and the leading producer in Africa, with 7.6 million bags in the 2020/21 calendar year (Melese & Kolech, 2021; USDA, 2021). It is the backbone of the country's economy and its chief export commodity (Hordofa & Tolossa, 2020). Ethiopia's coffee export alone accounts for 40% of the country's total exports, and in 2022, the country earned more than $1 billion (Ayele et al., 2021; Ethiopian Monitor, 2022).

Ethiopian Arabica specialty coffees are well‐liked because of their unique flavor profiles and consistently receive more than 90 points in international tastings (Alemu & Meijerink, 2010; Geeraert et al., 2019; Tolessa et al., 2019). A wide range of biochemical components, such as caffeine, trigonelline, chlorogenic acid, and sucrose, play a vital role in the exceptional flavor and aroma of the products (Tolessa et al., 2019). According to the literature, these well‐recognized qualities of Ethiopian Arabica coffees are attributed to their genetic diversity, favorable environmental conditions, and agro‐forestry‐based production system (Getachew et al., 2020; Girmay et al., 2019; Jima, 2020). Mainly, the altitude, soil fertility, and, most importantly, the optimum temperature of the country allowed the producers to produce specialty coffees (Tesfaye et al., 2020). Ethiopian specialty coffees from Harrar, Yirgacheffe, and Sidama have distinct flavor profiles (Alemu & Meijerink, 2010; Moat et al., 2017a, 2017b; Tolessa et al., 2019). Moreover, they earn a high value in the coffee market due to their exceptional flavor exhibited by their purity, geographical source, and consumers' demand for their origin (SCAA, 2014).

In recent years, certification of certain plants or plant‐derived products has emerged to inform consumers about their exceptional qualities due to their geographical origin (Abbas et al., 2018). Various certification methods inform consumers about the product's distinctiveness and protect it from fraud (Castro & Giraldi, 2018). The European Union introduced legislation presenting specific names for agricultural products, food, and beverages with distinctive quality characteristics under council regulation EEC N^o^. 2081/32, including the Protected Designation of Origin (PDO), Protected Geographical Origin (PGO), and Traditional Specialties are Guaranteed (TSG). These EU quality schemes were developed to protect the reputation of regional foods, benefit producers by earning a premium price for their authentic products, and minimize unfair and misleading computations from fraudulent products (1151/2012 EU regulation) (Maria‐Eleni & Apostolos, 2021).

Geographical indications (GIs) are a scheme under the World Trade Organization (WTO) and the Agreement on Trade‐Related Aspects of Intellectual Property Rights (TRIPS) (Kohsaka & Uchiyama, 2019). It is also used as a sign on products with a specific geographical origin that possesses qualities or a reputation due to that origin (UNCTAD, 2015). A product with certification of origin has twice the market value of a similar product without certification (Cassago et al., 2021). Moreover, the protection of GIs may encourage producers to safeguard the common quality standard of their products (UNCTAD, 2015). Registering for a geographical indication for a given agri‐food product requires demonstrating the link between the product's unique quality and the region where it is produced through traceability mechanisms. Traceability is the ability to provide information about a product's history and origin (Maria‐Eleni & Apostolos, 2021). The following section of this review article covers an overview of Ethiopian coffee production systems and foodomics technologies suitable for the traceability of Ethiopian Arabica coffees.

## 2 ETHIOPIAN ARABICA COFFEES AND THEIR PRODUCTION SYSTEM

In Ethiopia, over 5 million smallholder farmers are engaged in growing and producing coffee, and its production is growing steadily (Mas Aparisi, 2021; USDA, 2021). Coffee farming provides a source of income for 16% (15 million of Ethiopia's total population) in one way or another (Moat et al., 2017a, 2017b; Sisay, 2018). The country is a pool of genetic diversity for Arabica coffee, and coffee from different regions has distinct flavor profiles (Moat et al., 2017a, 2017b). The country's altitude, soil fertility, and optimum temperature allowed the producers to produce specialty coffee (Tesfaye et al., 2020). In the world coffee market, coffee quality is measured by the cupping score of the coffee. A coffee with a cupping score greater than 80 points based on the grading parameters of the Specialty Coffee Association of America (SCAA) is graded as a specialty coffee (Putri et al., 2019). Alternatively, Norwegian Erna Knutsen defined specialty coffee as coffee from a particular geographic microclimate with unique sensory profiles (Guimarães et al., 2020). Ethiopian specialty coffee from a single origin with exceptional flavor profiles from Harrar, Yirgacheffe, and Sidama has a premium price in the world coffee market (Alemu & Meijerink, 2010; Tolessa et al., 2019). The global demand for arabica specialty coffee is increasing; American adults' daily consumption increased from 14% in 2001 to 41% in 2017 (SCAA, 2017).

Harrar is an Ethiopian specialty coffee known for its medium‐sized, greenish‐yellow‐colored beans and creamy body with fruity characteristics. The coffee has a distinctive mocha flavor with medium acidity, which makes it exceptional (Sautier et al., 2018). The other internationally known Ethiopian specialty coffee is from Sidama regional state, called Sidama, and has a medium‐sized greenish or grayish‐colored bean. The coffee has fine acidity, a good body, a balanced taste with good flavor, and is identified as sweet coffee (Boot, 2011; Sisay, 2018). Sidama coffee has a wide range of flavors due to variations in soil type, microclimate, and countless heirloom coffee tree varieties across the region. It is always blended for specialty coffee (Sisay, 2018). Yirgacheffe is a microregion in the southern region that produces well‐known specialty coffee, internationally recognized under the brand name Yirgacheffe (Adane & Bewket, 2021; Boot, 2011; USAID, 2011). The coffee from the region has a fruity flavor with bright acidity and a silky mouthfeel (USAID, 2011). Moreover, the country is endowed with suitable environmental factors and a genetic pool for various Arabica coffee types with unique quality profiles, such as Harenna coffee from the Bale National Park and Amaro Kello coffee (Ascrizzi & Flamini, 2020; Garedew et al., 2022).

**FIGURE 1:**
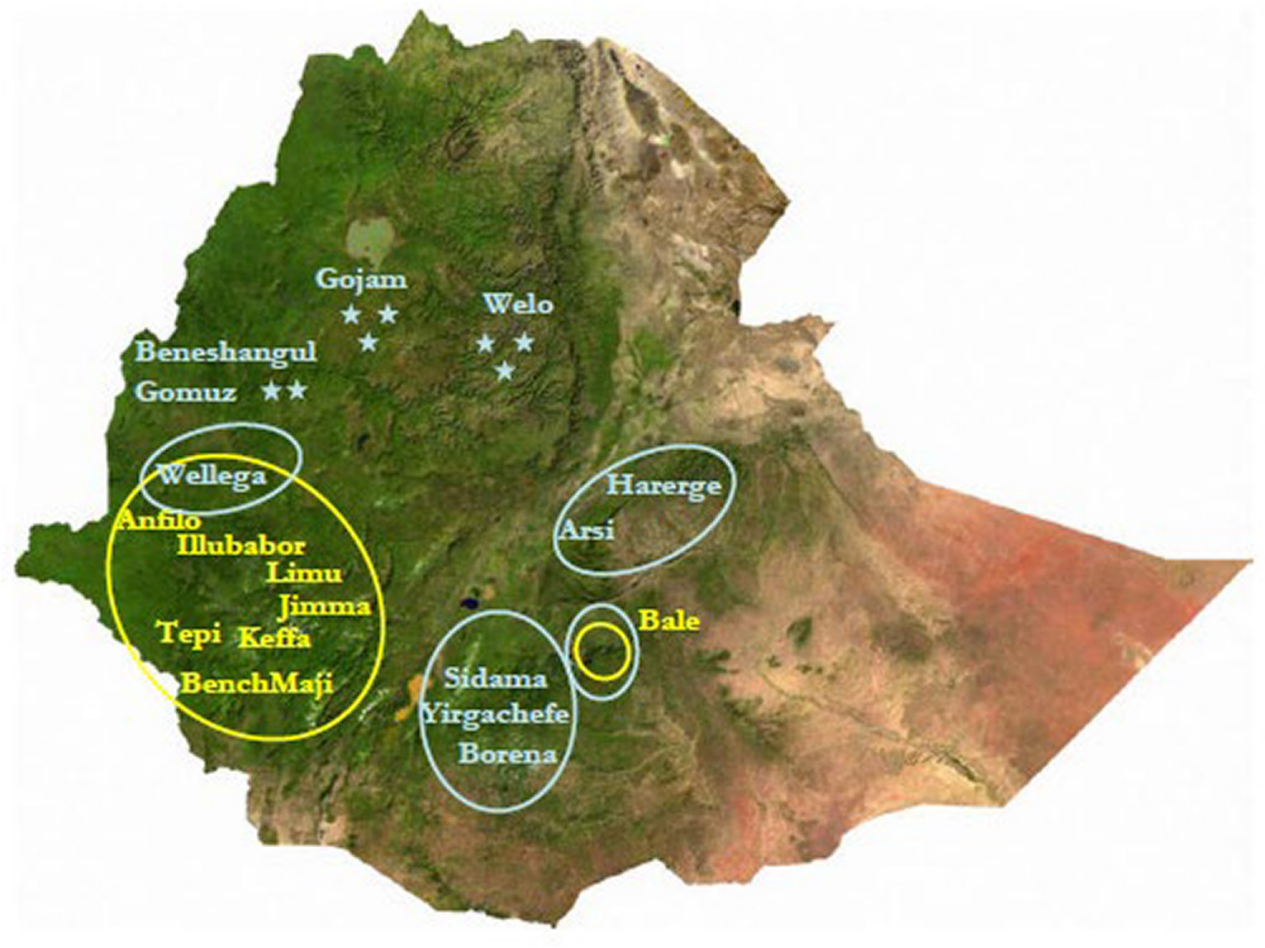
Main coffee‐producing areas of Ethiopia. Yellow circles are places where coffee is found in its natural habitat where it is picked in more or less managed forests as well as cultivated in gardens (blue circles and stars) (Labouisse & Kotecha, 2008).

In Ethiopia, coffee is produced organically based on structural complexity, vegetation, management level, and agronomic practices. The production systems are classified into four categories: forest coffee (FC) accounts for 10% of total production; plantation coffee covers 5%; semi‐managed forest coffee (SFC) holds 35%; and half (50%) of the total production system is garden coffee (GC) (Labouisse et al., 2008; Moat et al., 2017a; Muhie, 2022; Woyesa & Kumar, 2020). Different agroecological zones of the country are suitable for coffee production. Specifically, moist evergreen forests found at 650–2600 m above sea level are favorable for arabica coffee production (Friis et al., 2010; Muhie, 2022). As indicated in Figure 1 and Table 1, coffee is produced in the West, Southwest, Southern, Eastern, and Central regions of the country (Melkamu, 2015). In particular, the Oromia region, Sidama, and the Southern Nations, Nationalities, and Peoples Region (SNNPR) are major coffee producers. The Amhara region is a modest coffee producer, and Benishangul‐Gumuz is a minor coffee producer region (Moat et al., 2017a; Muhie, 2022).

**TABLE 1:** Main coffee‐growing region and their positions in Ethiopia.

Position in Ethiopia	Main coffee zone	Coffee area	Region	References
West of Rift Valley	North	*Amhara*	Amhara	Ango et al. (2020), Labouisse and Kotecha (2008), Melese and Kolech (2021), Moat et al. (2017a, 2017b)
		*Benishangul‐Gumuz*	Benishangul‐Gumuz	
	South West	*Wellega*	Oromia	
		*Illubabor*	Oromia	
		*Jimma‐Limu*	Oromia	
		*Tepi*	SNNPR and Gambela	
		*Kaffa*	SNNPR	
		*Bench‐Maji*	SNNPR	
Rift Valley	Rift	*Rift North*	SNNPR and Oromia	
		*Rift South*	SNNPR	
	South East	*Sidamo (including Yirgacheffe)*	Sidama and Oromia	
		*Bale*	Oromia	
		*Central Eastern Highlands*	Oromia	
East of Rift Valley	Harar	*Arsi*	Oromia	
		*West Hararge*	Oromia	
		*East Hararge*	Oromia	

## 3 ROLE OF ADVANCED FOODOMICS IN FOOD TRACEABILITY

The verification of food fairness and the elimination of fraudulent practices both depend on product authentication. The inaccuracy of food labels thus implies commercial deception, making food authentication a significant concern (Madesis et al., 2014). Both authenticity and traceability become the foundational elements of the food supply chain, which are essential parts of the system for defending food safety (Fanelli et al., 2021; González‐Domínguez, 2022). One of the most crucial factors in determining the authenticity of a food is its geographic origin, and agricultural products with geographical indications are known for their superior quality and distinctive regional characteristics (Zhao et al., 2022). In Europe, the primary concern with food authenticity is the origin of the food (Danezis, Tsagkaris, Brusic, et al., 2016). Recognition of the geographic origin of agri‐food products based on the organoleptic properties of the foods and the physicochemical properties of the product does not allow differentiation from similar products and they are not characteristics of geographic origin (Katerinopoulou et al., 2020).

Coffee is among the most adulterated foods. Mixing beans with different economic values is a standard method of coffee adulteration. As a result, the undeclared, and thus illegal, addition of cheaper robusta to arabica is regarded as a fraud (Klikarová & Česlová, 2022; Núñez et al., 2021). High‐quality single‐origin coffee producers may use misleading labels, false declarations, and fraudulent practices to increase their economic profit. The Swiss Federal Food Safety and Veterinary Office recently reported a falsely declared “100% Arabica” coffee that was substituted with the less expensive robusta coffee (Aurum et al., 2022; Federal Food Safety and Veterinary Office, 2019). Due to the fact that coffee quality is linked to specific growing areas, incorrect geographical indications are also illegal. In response to modern consumer demands for coffee origin authenticity, various strategies encompassing a wide range of technology and scientific techniques have been implemented to ensure (Aurum et al., 2022; Klikarová & Česlová, 2022).

A significant number of reliable and stable biomarkers necessitate identifying the authenticity of food, and the overall strategy is based on the following principles: Every organism (animal, plant, or microorganism) is characterized by its endogenous endowment (Creydt & Fischer, 2018). The DNA platform is used to encode endogenous processes, which are manifested as proteins (the proteome), which are then involved in the production and destruction of metabolic end products (the metabolome) (Creydt & Fischer, 2018; Selamat et al., 2021). This indicated genetic expression altered by external environmental factors (such as solar radiation and subsoil composition) or human activities (including pesticides, fertilizers, etc. applications). The alterations affect the sub‐molecular (elements and isotopes) profile over various timescales. The extent of exposure determines how much an external factor will affect a raw material. The pattern identifies the type of raw material and its source or origin (variety, provenance, environment, climate, and soil quality) and the type of cultivation (organic or conventional agriculture). The characteristics are made up of different elements, isotopes, and molecules and are comparable to the distinctiveness of a human fingerprint (Creydt & Fischer, 2018; Krajnc et al., 2021).

The specific chemical composition of the food (or its “fingerprint”) is a highly accurate predictor of its quality, origin, authenticity, and counterfeit. These fingerprints can vary based on the production processes, raw materials' origins, storage conditions, or adulteration techniques that have affected the levels of metabolites (Medina et al., 2019). In recent years, the authenticity of a variety of food commodities has been developed using omics‐based technologies, which are linked to the power of powerful and enormous molecular tools and can help get around the constraints of existing approaches (Balkir et al., 2021; Kafantaris et al., 2020; Zhao et al., 2022). In particular, foodomics uses a variety of omics technologies (genomics, transcriptomics, proteomics, and metabolomics) to offer molecular data on the various expression levels (i.e., gene, transcript, protein, or metabolite), as well as to integrate these data from a systems biology viewpoint (Afzaal et al., 2022; Rodríguez‐Carrasco, 2022; Theodoridis et al., 2021; Valdes et al., 2022). Recent reports have shown that foodomics techniques using tools from the proteomics, genomics, and metabolomics domains can provide precise and trustworthy traceability systems (Balkir et al., 2021; Valdes et al., 2022).

### 3.1 Proteomics

The proteome is a dynamic reflection of genes, transcripts, and the environment, and it has been used for biomarker discovery, identification, and validation of bioactive food peptides (Valdes et al., 2022). Proteomics is used to address some issues related to food authenticities, such as breed recognition, geographical origin, unclaimed addition of plant or animal protein material, type of material added (such as milk or milk proteins, blood or its constituents), production method (wild or farmed), and technological processing. Proteins are an indicator of food origin, properties, and processing (Afzaal et al., 2022; Chantada‐Vázquezab et al., 2022; Ignacio et al., 2016). Mainly, the proteomics technique is used in the authentication of hemoglobin and myoglobin in animal products, including those of pig, beef, sheep, and horse origin (Afzaal et al., 2022; Ignacio et al., 2016; López‐Pedrouso et al., 2020). In another way, electrophoretic protein profiles are mainly helpful in identifying very closely related aquatic species in seafood, such as fish and shellfish (Nissa et al., 2021).

Additionally, the method can be used for regional identification of samples that are high in protein. Nevertheless, more needs to be done to employ proteomic methods to determine the geographic origin of a particular food or food product (Zhang et al., 2018). The fundamental reason is that proteins are present in these samples only in trace amounts due to filtering processes used during industrial processing and the customary removal of the remaining proteins from the completed product. Moreover, very few proteins from the raw material withstand the fermentation process. They tend to cluster, resulting in sediments, so only small‐sized species of small fragments from the proteolysis of high‐molecular‐mass components are left following industrial modifications (Righetti et al., 2012).

The use of protein or peptide fingerprinting to detect fraud or authenticate origin, composition, variations, or vintage is further complicated by the presence of alien proteins (such as yeast, bacteria, and fungi). As a result, sample preparation is crucial and significantly impacts the number of proteins detected and the reproducibility of results (Ignacio et al., 2016). Proteomics, the comprehensive scientific study of all expressed proteins or the total proteome at any one time in an organism, complements genomics and transcriptomics techniques (Valdes et al., 2022).

### 3.2 Genomics

Genomics is the study of DNA for genetic traceability systems to identify living things and their products (Theodoridis et al., 2021). Due to the stability of DNA under environmental factors, farming practices, and production methods used during the processing of some food products, DNA‐based methods of plant species identification and authenticity of foods are thought to be a more accurate method for recognizing plant species, especially in food products (Madesis et al., 2014). One of the most commonly used methods of verification is molecular analysis, which may distinguish original (authentic) food goods from imitations (Danezis, Tsagkaris, Brusic, et al., 2016). This technique has been efficiently applied to the traceability of olive oil (Chedid et al., 2020; Marmiroli et al., 2009), breeds of sheep (Heaton et al., 2014), grapes, must, wine (Zambianchi et al., 2021), wheat and related cereals (Silletti et al., 2019), and coffee (Zhang et al., 2020).

Even though traditional polymerated chain reaction (PCR) techniques have been utilized for plant species identification, the methodology has a significant drawback. There needs to be more consistency among species. Additionally, there are a few limitations to the use of traditional PCR methods. The fundamental problem is that this technique is used in research settings. They rely on UV luminescence and the DNA double‐strand intercalation dye ethidium bromide to identify the amplification products during gel electrophoresis. Moreover, it is challenging to standardize the PCR process because it requires specific skills and careful handling to execute (from sample collection to result interpretation). Furthermore, the drawback of conventional PCR is that it cannot provide information on the quantitative analysis of the food product because its amplification products cannot quantify the original number of target molecules, which is particularly problematic in food products (Madesis et al., 2014).

Applying two or more omics approaches is required to go from theoretical assumptions to reliable and valuable results. For instance, genomics and transcriptomics alone cannot correctly describe the actions occurring within a cell; even when the information from DNA is transcribed to mRNA, proteins may not be biologically active (Ferrocino et al., 2022). Proteomics and genomics convey extensive data regarding the genotype but provide limited information about the phenotype. Conversely, metabolomics is the closest link between genotype and phenotype (Fiehn, 2002; Harrison et al., 2020).

### 3.3 Metabolomics

Metabolites are tiny molecules (less than 1500 Da) that come from various chemical classes (such as amino acids, carbohydrates, lipids, organic acids, and nucleotides). They are the byproducts of cellular regulatory processes due to numerous interactions among proteins, transcripts, and genes (Creydt & Fischer, 2018; Valdes et al., 2022). Thousands of different metabolites make up the metabolome. They are used to identify novel bioactive chemicals, determine the effects of agricultural methods, processing, and/or storage on the chemical composition of food, and ultimately conduct authenticity studies (Danezis, Tsagkaris, Camin, et al., 2016). Compared to other omics disciplines, the metabolome is most closely related to the phenotype and is more significantly influenced by exogenous influences, such as weather, soil composition, or storage conditions, than the proteomics (Class et al., 2021; Creydt & Fischer, 2018).

The metabolome, however, permits an even more apparent fingerprint of a system because numerous substances may be considered for evaluation, and the specific condition of a product at a specific time point can be predicted (Class et al., 2021). By assessing the total amounts of small‐molecule metabolites, metabolomics provides a picture of pertinent biological activities (amino acids, organic acids, starch, fatty acids, lipids, hormones, peptides, and vitamins). It offers a read‐on of the metabolic activity state for genetic variance, genetic expression, or outside influences. Additionally, metabolomics presents a picture of the host's physiology and its response to the environment, which might be tied to the outcome phenotype. They respond biologically to a variety of genetic and environmental cues. Metabolites differ in stability and have highly diverse turnover rates within cells (Selamat et al., 2021). Furthermore, metabolomics studies have revealed various novel metabolites and more specific biological traits for numerous species (Utpott et al., 2022). The metabolomics approach enables metabolic identification using analytical techniques like liquid or gas chromatography coupled with MS and directly assesses food's nutrient content (Selamat et al., 2021).

Among the foodomics approaches, metabolomics is reported as a precise and reliable tool for food traceability (Valdes et al., 2022). In addition, it involved quantitative and qualitative analyses of the whole set of metabolic processes (exogenous and endogenous metabolites, or metabolome) of a biological system under given environmental conditions (Djande et al., 2020; Jimenez‐Carvelo & Cuadros‐Rodrígue, 2021). The metabolome reflects the responses of the plant to physiological, pathophysiological, and environmental factors (Djande et al., 2020). Metabolomics is thus the metabolome science that correlates both the genotype and phenotype of a biological system that is altered by genetic and environmental changes (Brunetti et al., 2018).

Metabolomics is recommended as a potential geographical indication registration tool (Cassago et al., 2021). Especially in tracing the geographical origin of the food, the information gathered by metabolome analysis provides genuine information. Because the metabolome of the plant is very sensitive to environmental factors such as growing location, weather, or soil composition (Creydt et al., 2018), the metabolomic study requires incorporating analytical, spectrometric, and computational techniques to identify the distinctiveness of region‐specific agricultural products and foodstuffs (Ongo et al., 2020). In addition, metabolomics data analysis needs a sequential combination of one analytical technique and another to enrich the data. Mainly, it is associated with molecular diversity in terms of size, polarity, concentration level, and stability (Emwas et al., 2021).

Consequently, advanced high‐resolution analytical techniques with high separation capacity are required in metabolomics studies (Rodríguez‐Carrasco, 2022; Valdes et al., 2022). Some of the analytical instruments that are coupled and used for metabolite analysis include NMR coupled with gas chromatography (GC‐NMR), liquid chromatography (LC‐NMR), and solid‐phase extraction (LC‐SPE‐NMR). Liquid chromatography (HPLC) or ultra‐performance liquid chromatography (UHPLC) coupled with mass spectroscopy (LC–MS), HPLC‐MS, and UHPLC–MS have superior qualities over standard HPLC and LC. They utilize smaller bread in the column (<2 μm), operate under high pressure, and reduce the analysis cost (Emwas et al., 2021; Lu et al., 2008).

Headspace solid‐phase microextraction (HS‐SPME) coupled with GC–MS is another group of analytical tools used in food fingerprinting. The solid‐phase microextraction has high sensitivity, is simple to operate, and requires little or no sample preparation steps (Ch et al., 2021; Ongo et al., 2020). In some metabolomics studies, the liquid chromatography method coupled with time‐of‐flight mass spectrometry (ToF‐MS) (UPLC‐ToF‐MS) is used to obtain more accurate and precise MS data with good sensitivity and resolution to profile intact precursor ions (Qian et al., 2013). These combinations solve limitations of using a single analytical device and provide a synergistic effect (Emwas et al., 2021). Recent applications of the metabolomics technique with a chemometric data analysis approach in geographical indication have demonstrated the potential of metabolomics approaches to evaluate food traceability and investigate molecular changes due to geographical origin variation. Table 2 below summarizes an overview of metabolomics studies with their analytical techniques for geographical origin traceability.

**TABLE 2:** Overview of metabolomics studies with their analytical techniques in the geographical indication.

No.	Study	Analytical technique used	References
1.	Discrimination of geographical origin of agricultural products from small‐scale districts by widely targeted metabolomics with a case study on Pinggu peach	UPLC‐MS/MS (ultrahigh‐pressure liquid chromatography) coupled with a mass spectrometer and Qtrap mass spectrometer	Zhao et al. (2022)
2.	Comparative NMR metabolomics profiling between Mexican ancestral & artisanal mezcals and industrialized wines to discriminate geographical origins, agave species or grape varieties and manufacturing processes as a function of their quality attributes	NMR (nuclear magnetic resonance spectroscopy)	Lopez‐Aguilar et al. (2021)
3.	Metabolomic fingerprinting of volatile organic compounds for the geographical discrimination of rice samples from China, Vietnam, and India	HS‐GC–MS (headspace gas chromatography–mass spectrometry)	Ch et al. (2021)
4.	High‐performance liquid chromatography with fluorescence detection (HPLC‐FLD) fingerprints as chemical descriptors to authenticate the origin, variety, and roasting degree of coffee by multivariate chemometrics methods.	HPLC‐FLD (high‐performance liquid chromatography with fluorescence detection)	Nunez et al. (2021)
5.	Authentication of geographical origin in Hainan Partridge tea (*Mallotus oblongifolius*) by stable isotope and targeted metabolomics combined with chemometrics	LC–MS/MS (liquid chromatography coupled with double mass spectroscopy detector)	Fu et al. (2021)
6.	Non‐targeted LC–MS metabolomics approach toward an authentication of the geographical origin of grain maize (*Zea mays* L.) samples	LC–MS (liquid chromatography coupled with mass spectroscopy detector)	Schütz et al. (2021)
7.	Metabolomics fingerprint of Philippine coffee by SPME‐GC–MS for geographical and varietal classification	SPME‐GC–MS (headspace solid phase microextraction coupled with gas chromatography with mass spectroscopy detector)	Ongo et al. (2020)
8.	The volatile fingerprint of unroasted and roasted cocoa beans (*Theobroma cacao* L.) from different geographical origins	HS‐SPME‐GC–MS (headspace solid‐phase microextraction coupled with gas chromatography with mass spectroscopy detector)	Marseglia et al. (2020)
9.	Authentication of the geographical origin of extra‐virgin olive oil of the Arbequina cultivar by chromatographic fingerprinting and chemometrics	HPLC‐CAD (liquid chromatography coupled with charged aerosol detector) and HT‐GC‐FID (high‐temperature gas chromatography coupled with flame ionization detector)	Vera et al. (2019)
10.	Food targeting: geographical origin determination of hazelnuts (*Corylus avellana*) by LC‐QqQ‐MS/MS‐based targeted metabolomics application	LC‐QqQ‐MS/MS (liquid chromatography coupled with triple quadrupole mass spectrometry)	Klockmann et al. (2017)

## 4 AUTHENTICATION OF GREEN COFFEE THROUGH FOODOMICS APPROACH

Okubo and Kurata (2019) successfully classified green coffee bean samples from seven different countries: Cuba, Ethiopia, Indonesia (Bari, Java, and Sumatra), Tanzania, and Yemen. Zhu et al. (2021) compared the chemical and fatty acid composition of green coffee beans (*Coffea arabica* L.) from various geographical origins (China, Indonesia, Ethiopia, Kenya, Guatemala, Honduras, Brazil, and Colombia). The results revealed excellent discrimination between different geographical origins of green coffee beans, highlighting lipid, C24:0, C22:0, C18:3, C17:0, C18:0, C20:0, C16:0, and protein as discriminatory features. Miao et al. (2022) used an untargeted metabolomic approach based on UHPLC‐QE‐MS combined with fingerprint analysis to reveal an effective method for differentiating coffee beans from 18 green coffee bean samples sourced from Chinese importing companies and chosen to represent a variety of geographical origins. Likewise, Demianová et al. (2022) recently reported that arabica coffee volatiles correctly identified 100% of testing samples and predicted an accuracy of 86.96% in cross‐validation. Ketones, aldehydes, organic acids, esters, nitriles, alcohols, and alkenes explained 91.17% of the variability between African, South American, and Central American Arabica coffee samples, with ketones being the most potent parameter among volatiles. Pumbua et al. (2023) distinguished robusta coffees from various geographical locations and arabica coffees from various locations within the same province in Thailand with 80–100% accuracy.

## 5 RESEARCH PROGRESS IN GEOGRAPHICAL ORIGIN TRACEABILITY OF ETHIOPIAN SPECIALTY COFFEES

Profiling of the phenolic compounds for the geographical origin of green coffee beans from Ethiopia was conducted by Mehari, Redi‐Abshiro, Chandravanshi, Combrinck, Atlabachew, et al. (2016). They identified that 3‐caffeoylquinic acid, 3,4‐caffeoylquinic acid, 3,5‐caffeoylquinic acid, and 4,5‐caffeoylquinic acid were the most distinguishing chemicals for the authentication of different regional and sub‐regional green coffee beans. Similarly, the same group of researchers characterized the cultivation region of different Ethiopian Arabica coffee samples through elemental analysis. They reported phosphorous (P), manganese (Mn), copper (Cu), iron (Fe), and sulfur (S) as the most discriminant elements (Mehari, Redi‐Abshiro, Chandravanshi, Combrinck, & McCrindle, 2016). A study by Worku et al. (2019) used multi‐element and stable isotope profiling to differentiate the geographical origin of Ethiopian coffee from four different coffee‐growing regions of the country. XRF‐based multi‐elements with and without δ^13^C successfully discriminated the geographical origin of coffee with higher classification accuracy (89 and 86%, respectively) than ICP‐based multi‐elements with and without stable isotopes (80%, each) (Worku et al., 2019).

A study by Nunez et al. (2021) successfully differentiated the geographical origin of six coffee types from Colombia, Ethiopia, India, Indonesia, Nicaragua, and one unknown sample origin by using the metabolomics fingerprinting technique. Similarly, Ongo et al. (2020) employed a metabolomics approach in the geographical origin identification of arabica and robusta coffee in the Philippines through volatile metabolite analysis. In addition, metabolite profiling of *Coffea arabica* and *Coffea robusta* from different geographical origins in Indonesia (Putri et al., 2019). Endaye et al. (2020) investigated the geographical origin of green coffee Arabica beans from Ethiopia's Amhara regions and discovered that K, Mg, Ca, and Na were the main discriminators between samples. With an accuracy of 94.2% and a prediction ability of 93.4%, they classified coffee samples based on their production zones. Nevertheless, there needs to be more information on studies that report on the geographical origin traceability of Ethiopian Arabica specialty coffee through metabolomic study.

## 6 CRITICAL ANALYSIS AND COMPARISONS

Regional foods' reputation helps producers earn premium prices for their authentic products and protects consumers from unfair and misleading competition from fraudulent products (Maria‐Eleni & Apostolos, 2021). Moreover, the product quality associated with its geographic origin becomes a significant criterion for consumers to purchase the product (Teye et al., 2019). Geographical designations can be protected by trademarks (TMs) or geographical indicators (GIs), both of which have different benefits and costs in different settings (Schubler, 2009). So far, Ethiopian Yirgacheffe, Sidamo, and Harar coffees have registered trademarks in key international markets. However, it does not benefit the country like those geographical indications do since a product with certification of origin has twice the market value of a product without certification (Cassago et al., 2021).

Additionally, Ethiopia faced challenges for different agri‐food products' intellectual property rights, such as the dispute between the known coffee company Starbucks and the Ethiopian government to acquire the trademark for the vital export market of Ethiopian specialty coffees (Yirgacheffe, Sidamo, and Harar) (Arslan & Christopher, 2010). Moreover, more recently, the right to the intellectual property of teff (*Eragrosris tef*) was also raised (Andersen & Winge, 2012; Simmonds et al., 2020). Consequently, protecting geographical origin necessitates Ethiopian specialty coffee because increased demand for specialty coffee in the international market dictates differentiating its origin (Giovannucci et al., 2008). It is necessary to implement intellectual property tools to ensure certification of single‐origin coffee in producing countries.

Furthermore, Ethiopia is known for being the center of origin for various agricultural products, including coffee; it is the only country in the world where coffee plants still grow wild. According to the United Nations Conference on Trade and Development (UNCTAD, 2015), Ethiopia has potential products eligible for geographical indication registration. Moreover, UNCTAD indicated the need for urgent advertising and establishing branding for Harenna wild coffee, Wenchi volcanic honey, and Wukro honey. Even though the country drafted a geographical indications proclamation and GI regulation in 2012, the GI law has not yet been implemented. Even though the geographical indication registration process is time‐consuming, demanding, bureaucratic, and requires collective investment and efforts (Quinones‐Ruiz et al., 2016), it has a significant impact on the market value of the product and has the advantage of creating new market opportunities for the product and benefits the actors (Rupprecht et al., 2020). Consequently, the UNCTAD emphasized that Ethiopia's public and private sectors improve traceability mechanisms throughout the nation's coffee‐growing regions (UNCTAD, 2015).

Foodomics technology is an emerging area of science that is widely applicable in various research areas in different parts of the world for the traceability of regional foods via volatile aroma compound metabolomics fingerprinting, such as Philippine coffee (Ongo et al., 2020), Hainan Partridge tea (Fu et al., 2021), rice from China, Vietnam, and India (Ch et al., 2021), and many other products. However, in Ethiopia, the discipline is applied little in research and is in its infancy state. These technologies are reported as a suitable approach for the traceability of food products. In particular, the metabolomics approach is reported as the appropriate method to provide concrete evidence regarding the association between the exceptional qualities of agri‐food products and their geographical origin. Table 3 depicts an overview of the strengths and limitations of omics technologies.

**TABLE 3:** The strength and limitations of Omics technologies for food traceability.

Omics techniques	Strengths	Limitations	References
Genomics	Highly specific It is more precise Quick Suitable for analysis of a large sample size	Provides limited information about the phenotype Interpretation of genome and transcriptome data with biological function, that is, how particular polymorphisms affect phenotypic variation	Dimitrakopoulou et al. (2021), Misra et al. (2019), Namin et al. (2022)
Proteomics	Fast and provides in‐depth analysis of food at the peptide level It is used as a more rapid, flexible, and high‐throughput approach for evaluating the authenticity and traceability of species in food items	Provides limited information about the phenotype Due to the lack of protein amplification, proteomics methods need substantial volumes of samples Lack method of validation, standardization, and most importantly the analysis is complex	Afzaal et al. (2022), Misra et al. (2019)
Metabolomics	Closest to the phenotype and link both the genotype and phenotype Linking the genome, transcriptome, and proteome to phenotype provides a crucial tool for identifying the genetic basis of phenotype	Like proteins, metabolites are not amplifiable and only 15–30% of the entire mass spectra are identifiable and quantifiable	Harrison et al. (2020), Misra et al. (2019)

## 7 CONCLUSION AND FUTURE REMARKS

The general overview of the Ethiopian Arabica coffee production system and the role of food omics techniques in the reputation of its geographical origin were addressed. Even though various studies address Ethiopian specialty coffees and their exceptional qualities, there needs to be more scientific work on the chemical compounds behind their unique taste and flavor and the traceability of their geographical origin. Consequently, the benefit of single‐origin products is not exploited due to a lack of reliable traceability information about their geographical origin. According to the literature, the recently emerged foodomics technologies are reported as suitable means of product authentication and traceability. However, the science of omics technology is also in its infancy. It has yet to come up with concrete evidence that helps to elucidate the link between Ethiopian Arabica coffee quality and its geographical origin. There is a paucity of studies on the geographical origin indication of Ethiopian coffees through foodomics traceability techniques. Nevertheless, a robust metabolomics‐based traceability system that provides evidence of the association between the exceptional qualities of Ethiopian coffees and their geographical origin is recommended.

## AUTHOR CONTRIBUTIONS

**Makiso Urugo Markos:** Conceptualization (equal); investigation (equal); methodology (equal); writing – original draft (equal). **Yetenayet Tola:** Investigation (equal); methodology (equal); supervision (equal); writing – review and editing (equal). **Biniam T. Kebede:** Methodology (equal); supervision (equal); writing – review and editing (equal). **Onwuchekwa Ogah:** Methodology (equal); supervision (equal); writing – review and editing (equal).

## ACKNOWLEDGEMENTS

The authors would like to thank every individual who contributed their part to the successful development of this article.

## FUNDING INFORMATION

This research did not receive any specific grant from funding agencies in the public, commercial, or not‐for‐profit sectors.

## CONFLICT OF INTEREST STATEMENT

The authors declare that they have no conflict of interest.

## ETHICS STATEMENT

No ethical approval was required as this is a review article with no original research data.

## Data Availability

The data that support the findings of this study are available from the corresponding author upon reasonable request.
